# Efficacy of Endoscopic Ultrasound‐guided Transluminal Drainage Using Lumen‐apposing Metal Stents for the Treatment of Pancreatic Fluid Collections

**DOI:** 10.1002/deo2.70249

**Published:** 2025-11-22

**Authors:** Keisuke Kinoshita, Kazuhisa Okamoto, Haruna Noguchi, Satoshi Fukuchi, Hidetoshi Akiyama, Mitsuteru Motomura, Yoshifumi Azuma, Yasuhisa Hiroshima, Takafumi Fuchino, Sotaro Ozaka, Ryota Sagami, Takuro Uchida, Yuka Hirashita, Kensuke Fukuda, Ryo Ogawa, Kazuhiro Mizukami, Masaaki Kodama, Kazunari Murakami

**Affiliations:** ^1^ Department of Gastroenterology Faculty of Medicine Oita University Oita Japan; ^2^ Department of Gastroenterology Oita City Medical Association Almeida Memorial Hospital Oita Japan; ^3^ Department of Gastroenterology Oita Red Cross Hospital Oita Japan

**Keywords:** double‐pigtail plastic stent, endoscopic ultrasound‐guided transluminal drainage, lumen‐apposing metal stent, pancreatic fluid collections, walled‐off necrosis

## Abstract

**Objectives:**

Pancreatic fluid collections (PFCs) are able to develop secondary to either fluid leakage or liquefaction of acute pancreatitis, chronic pancreatitis, pancreatic trauma, or after pancreatic surgery. While most PFCs resolve spontaneously, endoscopic procedures are sometimes necessary. Endoscopic ultrasound‐guided transluminal drainage (EUS‐TD) using lumen‐apposing metal stents (LAMS) is now widely performed for PFCs. This study investigated the incidence of complications and points to be considered during and after EUS‐TD using LAMS for the treatment of patients with PFCs.

**Methods:**

This three‐center retrospective study investigated patients who underwent EUS‐TD using LAMS or using a double‐pigtail plastic stent (DPS) for the treatment of PFCs at the University of Oita Hospital and affiliated institutions from December 2018 to June 2024. The primary outcome was the clinical success rate. Secondary outcomes included the technical success rate, endoscopic procedure time, LAMS indwell term, adverse events (AEs), and post‐LAMS placement course.

**Results:**

Twenty‐five patients (mean age, 67.5 ± 15.4 years; 76% male) underwent LAMS placement for PFCs. Median size of PFCs was 90 mm (range, 40–227 mm). A clinical success rate was achieved in 96%. The technical success rate was 96%, and the median endoscopic procedure time was 11 min (range, 5–32 min). Median LAMS indwell term was 46 days (range, 36‐60 days). AEs were bleeding and stent migration. Post‐LAMS placement course was good in 22 patients. No patients required re‐treatment after LAMS removal.

**Conclusions:**

EUS‐TD using LAMS is a simple procedure for the treatment of PFCs and appears clinically effective, highly safe, and efficient compared to conventional EUS‐TD using DPS.

## 1 Introduction

Pancreatic fluid collections (PFCs) are able to develop secondary to either fluid leakage or liquefaction in acute pancreatitis, chronic pancreatitis, pancreatic trauma, or after pancreatic surgery. Among PFCs, pancreatic pseudocysts (PPC) and walled‐off necrosis (WON) that occur more than 4 weeks from the onset of acute pancreatitis are good indications for interventional endoscopic ultrasound procedures using a lumen apposing metal stent (LAMS) [1, 2]. However, recent data have raised concerns regarding the potential for delayed adverse events (AEs) associated with the indwelling LAMS. Indeed, several studies have reported delayed bleeding and buried stent syndrome, in which extensive mucosal tissue overgrowth around the proximal flange on the luminal side compromises stent removal during follow‐up [3, 4, 5, 6, 7, 8]. Surgical interventions have been used as conventional treatments in the treatment of PFCs, but are associated with high rates of AEs and mortality. A recent meta‐analysis reported that the endoscopic step‐up approach was associated with significantly shorter hospital stays and lower rates of complications such as multiple‐organ failure, perforation, enterocutaneous, and pancreatic fistula than methods applying a surgical step‐up approach [9, 10, 11]. The endoscopic step‐up approach is therefore the first choice of treatment for PFCs. Recently, the technique of placing LAMS gained coverage by insurance for the first time in Japan, starting from September 2018, and endoscopic ultrasound‐guided transluminal drainage (EUS‐TD) using a LAMS is now widely performed for PFCs [12, 13, 14, 15]. The present study investigated the incidence of complications and points of consideration during and after EUS‐TD using LAMS for the treatment of patients with PFCs.

## 2 Materials and Methods

This was a three‐center, retrospective study of EUS‐TD using LAMS or using a double‐pigtail plastic stent (DPS) for the treatment of PFCs performed by five experienced endoscopists in Oita from December 2018 to June 2024. This study was approved by the ethics committee of Oita University Hospital (approval no. 2773). Written informed consent was obtained from all patients for publication.

### 2.1 Patients

Consecutive patients who underwent EUS‐TD using LAMS or DPS for the treatment of PFCs at the University of Oita Hospital and affiliated institutions from December 2018 to June 2024 were included. Only symptomatic PFCs were included in this study. PFCs were defined as a collection of mature encapsulations surrounding pancreatic necrosis, measuring 4 cm or larger, with a distinct inflammatory wall and at least 70% fluid content. Underlying pathologies in patients with PFCs included severe acute pancreatitis (SAP), alcoholic pancreatitis, obstructive pancreatitis due to malignancy, idiopathic pancreatitis, traumatic pancreatitis, post‐endoscopic retrograde cholangiopancreatography (ERCP) pancreatitis, and postoperative pancreatic fistula. The inclusion criteria are as follows: (1) refractory abdominal pain; (2) biliary obstruction; (3) PFCs size ≥4 cm; (4) fully encapsulated PFCs with ≥70% fluid content; (5) absence of intervening vessels. The exclusion criteria are as follows: (1) coagulation disorders (INR >1.5); (2) asymptomatic PFCs; (3) disconnected pancreatic duct syndrome; (4) PFCs where the combined thickness of the gastrointestinal and cyst walls is ≥10 mm to the EUS probe; (5) presence of intervening vessels. There were no selection criteria for LAMS versus DPS, nor any facility protocols guiding this decision; the choice of which to use was determined by the operator's preference at each facility.

### 2.2 LAMS Placement Procedures

In this study, all procedures were performed using a therapeutic linear echo‐endoscope with a working channel of 4.2 mm (GF‐UCT180; Olympus America, Centre Valley, PA, USA) under local anesthesia. EUS examination of patients with PFCs was performed to assess for the presence of necrotic material. Once an appropriate site was identified from the stomach or duodenum, we used the LAMS (Hot AXIOS; Boston Scientific Corporation, Marlborough, MA, USA). The initial puncture was performed, and the cyst was accessed. Then, by sequentially unlocking and locking individual components of the delivery system, the intra‐cystic flange of the stent was deployed first. This flange was then approximated against the cyst wall and followed by the release of the intra‐luminal end within the endoscope. At this point, by simultaneously pushing the stent outwards from the endoscope channel and moving the endoscope away from the mucosal surface, the stent was completely deployed. Stent placement was then verified endoscopically.

### 2.3 DPS Placement Procedure

In this study, all procedures were performed using a therapeutic linear echo‐endoscope under local anesthesia. Previously, an EUS examination was performed to assess for the presence of necrotic material and the local vasculature and determine the puncture site. Primary puncture of the PFC cyst cavity was performed using a 19‐gauge needle. Subsequently, contents were aspirated, contrast medium was injected to confirm positioning, and a 0.035‐inch guidewire was inserted through the needle and coiled within the PFCs. The needle was then removed, leaving the guidewire in the cyst. The pathway was subsequently dilated using an ES dilator (DC7R180S; Zeon Medical Co., Ltd., Tokyo, Japan) and a controlled radial expansion wire guide balloon with a diameter of 4 mm, 6 mm. For patients in whom the PFCs had been drained using a DPS, two 7Fr DPS of 7 and 10 cm were placed within the PFCs alongside the wire, under endoscopic and fluoroscopic guidance.

### 2.4 Outcome Parameters

The primary outcomes were (1) the clinical success, defined as resolution of PFCs symptoms or at least 50% reduction in the size of PFCs following LAMS or DPS insertion; and (2) the incidence of complications. Secondary outcomes included: the technical success rate, defined as the rate of successful LAMS or DPS placement; endoscopic procedure time, calculated as time from scope in to scope out; LAMS or DPS indwell term, counted from initial LAMS or DPS placement until removal; AEs, defined as occurring within 30 days after initial LAMS or DPS placement procedure; and post‐LAMS or DPS placement course.

### 2.5 Statistical Analysis

The Mann–Whitney *U* test and Fisher's exact probability test were used to analyze differences in this study. Data statistics analysis was performed with Statistical Package for the Social Sciences software (SPSS Statistics version 28; SPSS, Tokyo, Japan). Data was reported as median and interquartile range. Values of *P*<0.05 were considered indicative of a statistically significant difference.

## 3 Results

### 3.1 Characteristics of Patients With PFCs and Technical Procedures

**FIGURE 1:**
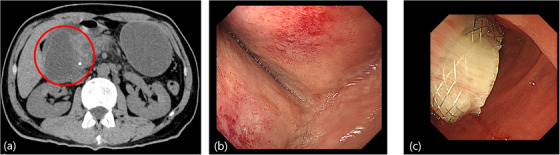
Preoperative case of pancreatic head cancer with duodenal bulb route of lumen‐apposing metal stents (LAMS) placement. (a) The patient was admitted to our hospital with vomiting and anorexia. The patient had undergone insertion of a plastic stent into the common bile duct because of obstructive cholangitis caused by cancer of the pancreatic head. Contrast‐enhanced computed tomography revealed obstructive pancreatitis due to the pancreatic head cancer and pancreatic fluid collections (red circle) on the tail side of the pancreatic head cancer. (b) Upper endoscopy revealed extrinsic compression by pancreatic fluid collections of the pancreatic head in the duodenal bulb. The endoscope encountered difficulty passing to the second part of the duodenum, revealing a passage obstruction. (c) In this case, due to the preoperative state of pancreatic head cancer, the site of LAMS placement was selected using the duodenal bulb route, which is within the scope of subtotal stomach‐preserving pancreaticoduodenectomy.

**FIGURE 2:**
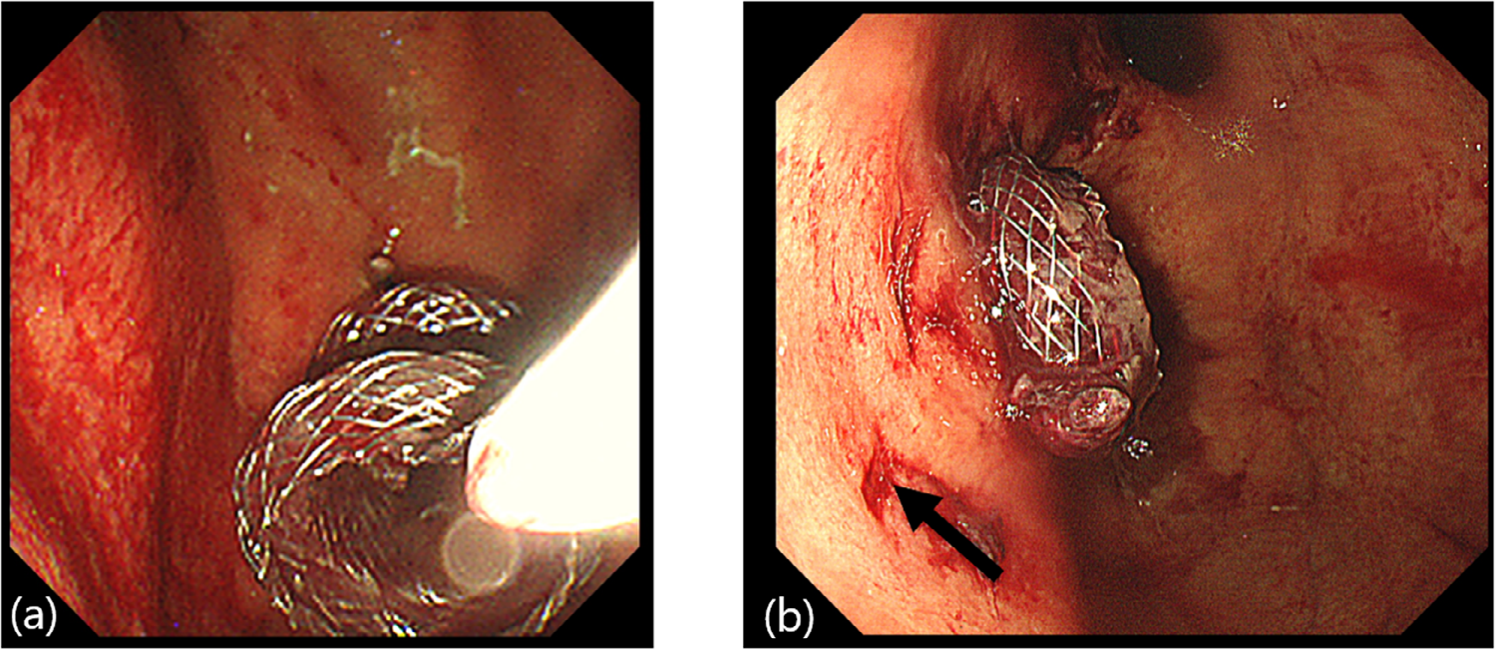
Adverse events of mis‐development during lumen‐apposing metal stents (LAMS) insertion. (a) Endoscopic findings in the case with LAMS mis‐deployment. The distal flange of the LAMS was pulled out to the stomach during LAMS deployment. (b) A new LAMS is inserted into the walled‐off necrosis (WON) of the pancreatic tail. A puncture hole (arrow) is seen, representing the first LAMS insertion site, on the anal side of the new LAMS.

Characteristics of patients with PFCs treated using LAMS included 12 cases of SAP (including four cases of alcoholic pancreatitis), five cases of obstructive pancreatitis due to acute lymphoblastic leukemia with B‐cell precursor phenotype or pancreatic head cancer or hematoma, two cases of idiopathic pancreatitis, one case of traumatic pancreatitis, two cases of post‐ERCP pancreatitis, three cases of postoperative pancreatic fistula, 18 cases of PPC, and seven cases of WON. In contrast, the characteristics of PFC patients treated with DPS included nine cases of SAP (including five cases of alcoholic pancreatitis), two cases of obstructive pancreatitis due to pancreatic head cancer, two cases of post‐ERCP pancreatitis, six cases of postoperative pancreatic fistula, 20 cases of PPC, and six cases of WON (Table 1). Characteristics of technical procedures and clinical outcomes are summarized in Table 2. Median size of PFCs treated using LAMS or DPS was 90 mm (range, 40–227 mm), 69 mm (range, 41–166 mm), respectively. Twenty‐two of the 25 cases used LAMS with a diameter of 15 mm. The remaining three cases used a 10‐mm LAMS. LAMS placement for the treatment of PFCs was technically successful in all except one case (24/25, 96%). In contrast, DPS placement for the treatment of PFCs was technically successful in all cases (26/26, 100%). Among PFCs treated with LAMS or DPS, technical success was achieved in all 18 cases (100%) and all 20 cases (100%), respectively, for PPC, and in six of seven cases (86%) and all six cases (100%), respectively, for WON. The site of LAMS placement was commonly selected at the proximal stomach route (fundus, cardia, or gastric body), except for one case in which the duodenal bulb route was selected due to preoperative status for cancer of the pancreatic head (Figure 1). One case involved LAMS misdevelopment during the endoscopic procedure for LAMS placement (Figure 2). Median endoscopic procedure time of the treatment of PFCs using LAMS was 11 min (range, 5–32 min), significantly shorter than conventional treatment of PFCs using DPS (22 min; range, 17–64 min) (Table 2).

**TABLE 1:** Characteristics of patients with pancreatic fluid collections (PFCs).

	LAMS (*n* = 25)	DPS (*n* = 26)
Age, mean (SD), years	67.5 (15.4)	60.4 (12.9)
Sex, male/female, n (%)	19 (76)/6 (24)	24 (92)/2 (8)
Performance status, *n* (%)		
PS 0	0 (0)	0 (0)
PS 1	4 (16)	12 (46)
PS 2	13 (52)	12 (46)
PS 3	6 (24)	1 (4)
PS 4	2 (8)	1 (4)
Patients admitted to the ICU, *n* (%)	2 (8)	1 (4)
Etiology of PFCs, *n* (%)		
Severe acute pancreatitis	12 (48)	9 (35)
Alcoholic pancreatitis	4 (16)	16 (61)
Obstructive pancreatitis	5 (20)	2 (8)
Idiopathic pancreatitis	2 (8)	0 (0)
Traumatic pancreatitis	1 (4)	0 (0)
Post‐ERCP pancreatitis	2 (8)	2 (8)
Postoperative pancreatic fistulas	3 (12)	6 (23)
Walled‐off necrosis	7 (28)	6 (23)
Pancreatic pseudocyst	18 (72)	20 (77)
PFCs location, *n* (%)		
Head of pancreas	2 (8)	1 (4)
Body of the pancreas	15 (60)	8 (31)
Tail of the pancreas	8 (32)	17 (65)

Abbreviations: DPS, double‐pigtail plastic stent; ERCP, endoscopic retrograde cholangiopancreatography; ICU, intensive care unit; LAMS, lumen‐apposing metal stent; PFCs, pancreatic fluid collections; PS, performance status; SD, standard deviation.

**TABLE 2:** Characteristics of technical procedures and clinical outcomes.

	LAMS (*n* = 25)	DPS (*n* = 26)	*p*‐value
PFCs size, median (IQR), mm	90 (65–108)	69 (64–132)	0.71
Diameter of LAMS, *n* (%)			
10 mm	3 (12)		
15 mm	22 (88)		
Site of stent placement, *n* (%)			
Transgastric route	24 (96)	25 (96)	0.98
Transduodenal bulb route	1 (4)	1 (4)	0.98
Technical success, %	96	100	0.33
PPC case	100	100	
WON case	86	100	1
Clinical success, %	96	88.5	0.32
PPC case	100	90	0.49
WON case	86	83	1
Procedure time, median (IQR), min	11 (5–22)	22 (19–39)	<0.01
Cases of performed DEN, *n* (%)	4 (16)	0 (0)	0.04
Stent indwell term, median (IQR), days	46 (36–60)	282 (158–450)	<0.01
Adverse events, *n* (%)	3 (12)	5 (19)	0.49
Recurrence, *n* (%)	0	0	NA

Abbreviations: DEN, direct endoscopic necrosectomy; DPS, double‐pigtail plastic stent; IQR, interquartile range; LAMS, lumen‐apposing metal stent; NA, not applicable; PFCs, pancreatic fluid collections; PPC, pancreatic pseudocyst; WON, walled‐off necrosis.

### 3.2 Clinical Outcomes

**FIGURE 3:**
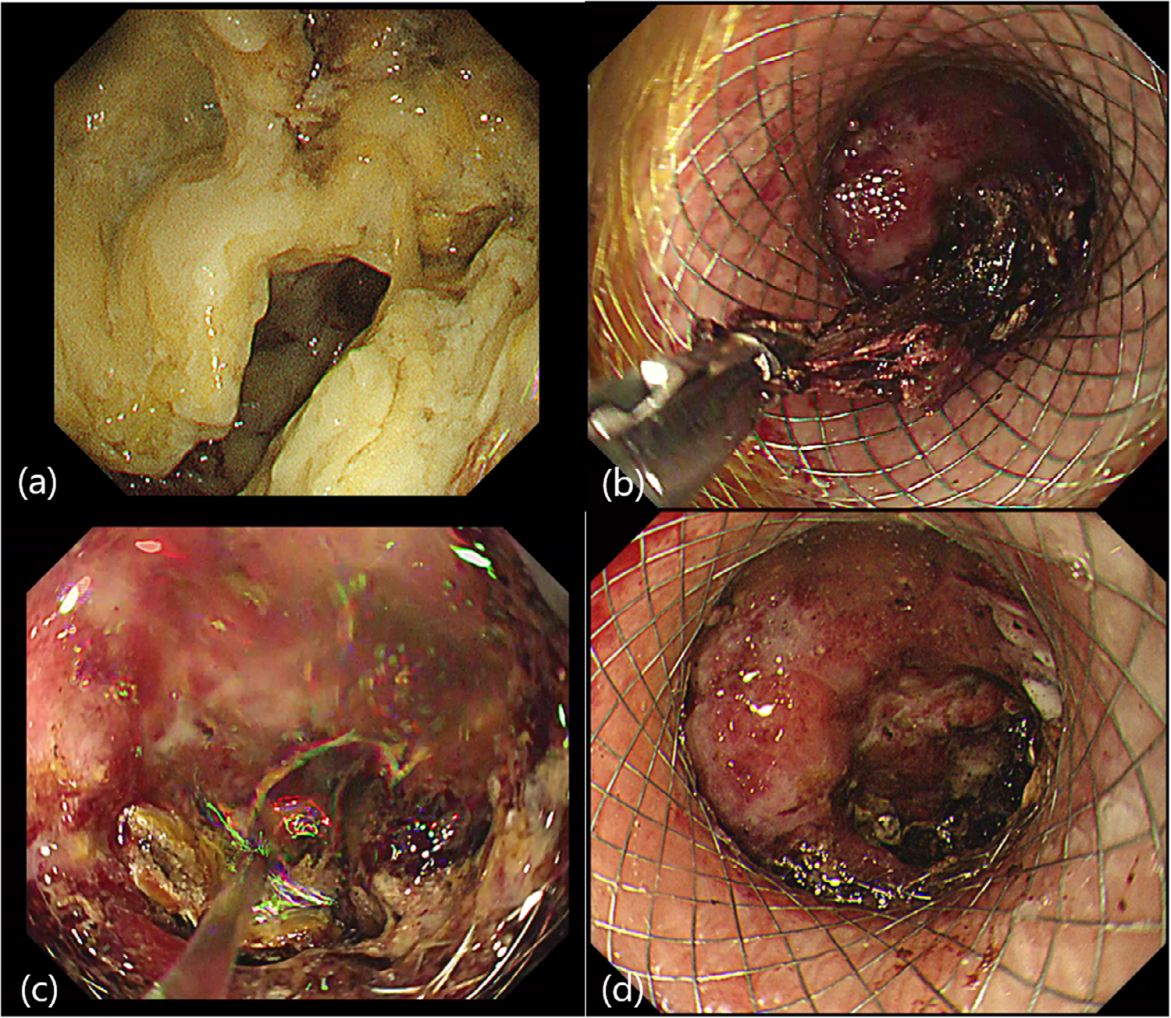
Direct endoscopic necrosectomy (DEN) after lumen‐apposing metal stents (LAMS) placement. (a) Endoscopic findings before DEN reveal the inside of the pancreatic fluid collections (PFCs) filled with a large amount of necrotic tissue. (b) Endoscopic findings during DEN with biopsy forceps. (c) Endoscopic findings during DEN with the endoscopic waterjet device. (d) Endoscopic findings after DEN reveal that the inside of the PFCs is clear.

Characteristics of primary outcomes and clinical success are summarized in Table 2. Clinical success of LAMS placement for the treatment of PFCs was achieved in 24 of 25 cases (96%). In contrast, clinical success of DPS placement for the treatment of PFCs was achieved in 23 of 26 cases (88.5%). Among PFCs treated with LAMS or DPS, clinical success was achieved in all 18 cases (100%) and 18 of 20 cases (90%), respectively, for PPC, and in six of seven cases (86%) and five of six cases (83%), respectively, for WON. The criteria for LAMS removal included resolution of PFC symptoms or at least 50% reduction in the size of PFCs on imaging findings. Median LAMS indwell term was 46 days (range, 36–60 days). Four cases underwent direct endoscopic necrosectomy (DEN) after LAMS placement for the treatment of PFCs (Figure 3). In all cases, PFCs tended to shrink after DEN, and the LAMS could be removed, and no patients experienced recurrence after LAMS removal. In the other cases, PFCs shrank with LAMS placement alone for the treatment of PFCs and did not require further treatment. No cases required additional percutaneous drainage or surgery.

### 3.3 Incidence of Complications and AEs

**FIGURE 4:**
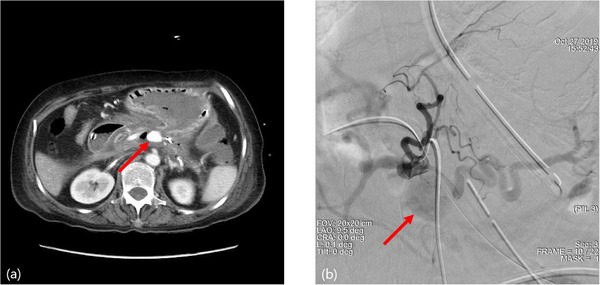
Adverse events of splenic pseudoaneurysm rupture after lumen‐apposing metal stents (LAMS) placement. (a) Eighty days after LAMS placement, the patient experienced massive hematemesis and fell into shock. Contrast‐enhanced computed tomography revealed that a splenic pseudoaneurysm (red arrow) had developed after LAMS placement. (b) Interventional radiology confirmed a splenic pseudoaneurysm (red arrow). The patient underwent hemostasis by embolization with interventional radiology.

Overall, very few AEs developed, occurring in only three of the 25 cases in the LAMS group and five of the 26 cases in the DPS group. There was no significant difference in the incidence of complications between the two groups. One case had spontaneous stent migration. The other cases showed bleeding caused by splenic pseudoaneurysm rupture after LAMS placement, and hemostasis was achieved in these patients using interventional radiology (Figure 4a,b). The five cases in the DPS group involved stent migration, descending colon perforation by the stent, intra‐cystic hematoma, sepsis, and peritonitis. Each case improved with follow‐up observation, antibiotic administration, or systemic management via continuous renal replacement therapy. No cases of buried stent syndrome, LAMS occlusion, perforation, delayed AEs, or LAMS placement procedure‐related mortality were seen in this study.

### 3.4 LAMS Removal and Clinical Follow‐up

Follow‐up endoscopy after LAMS placement for the treatment of PFCs was performed at a median of 7 weeks (range, 3–12 weeks). However, assessment of clinical outcomes was not possible for three patients who were lost to follow‐up due to transfer to another hospital or because the family did not wish to have the LAMS removed or more endoscopies performed. Although three cases of in‐hospital mortality due to worsening pancreatitis in the LAMS group (two cases of SAP and one case of traumatic pancreatitis) and four cases in the DPS group (all cases of SAP), all patients who could be evaluated for clinical outcomes after LAMS or DPS removal showed good status without recurrence of PFCs and no need for further treatments.

## 4 Discussion

In this study, we confirmed that EUS‐TD using LAMS is useful for the treatment of patients with PFCs and that the LAMS placement procedure for patients with PFCs is simple, highly safe, and clinically effective. EUS‐TD is now the standard treatment for the initial management of symptomatic PFCs. EUS‐TD offers similar efficacy to surgery, with shorter recovery times, a lower adverse event rate, and improved cost‐effectiveness [9, 11]. Dedicated LAMS have recently been developed to simplify endoscopic procedures for PFC drainage. The LAMS was first reported by Binmoeller and Shah in 2011, and was designed for transintestinal luminal drainage [16]. Recently, the development of LAMS devices has further consolidated this procedure by overcoming some of the inherent technical limitations associated with the use of conventional DPS and fully covered self‐expanded metal stents [17]. However, despite accumulating data on the efficacy and safety of LAMS placement for PFCs drainage, concerns regarding the risk of delayed AEs associated with LAMS placement for PFCs drainage have been increasing. Even so, Yang et al. demonstrated that EUS‐TD using LAMS placement for the treatment of patients with PFCs is effective and safe, delayed AEs are rare, and the use of electrocautery‐enhanced LAMS was the sole predictor of failure of WON resolution in their multicenter study [18].

Symptoms of PFCs, such as abdominal pain, fever, or signs of infection, are indications for treatment with drainage. Regarding the timing of treatment for patients with PFCs, intervention 4 weeks after the onset of acute pancreatitis has been recommended because incomplete encapsulation results in higher complication and mortality rates [1]. In this study, we treated completely encapsulated PFCs that occurred more than 4 weeks after the onset of acute pancreatitis, with no intervening vessels, a combined thickness of gastrointestinal and cyst wall less than 10 mm to the EUS probe, and at least 70% fluid component. We therefore considered that EUS‐TD using LAMS for the treatment of patients with PFCs in this study was performed safely and encountered no deaths related to EUS‐TD procedures using LAMS. While no consensus has been reached on the management of asymptomatic PFCs, current guidelines suggest conservative treatment, regardless of the size of PFCs. Although the optimal timing of treatment for patients with asymptomatic PFCs is very difficult to determine, Kumar et al. evaluated outcomes in 30 consecutive patients with asymptomatic WON associated with acute pancreatitis and recommended conservative management for those patients. They reported that about one‐quarter of asymptomatic patients with WON developed infection, although management with minimally invasive interventions such as percutaneous or endoscopic drainage was successful, with no mortality [19]. However, further randomized, prospective controlled trials are now needed to provide definitive insights.

**FIGURE 5:**
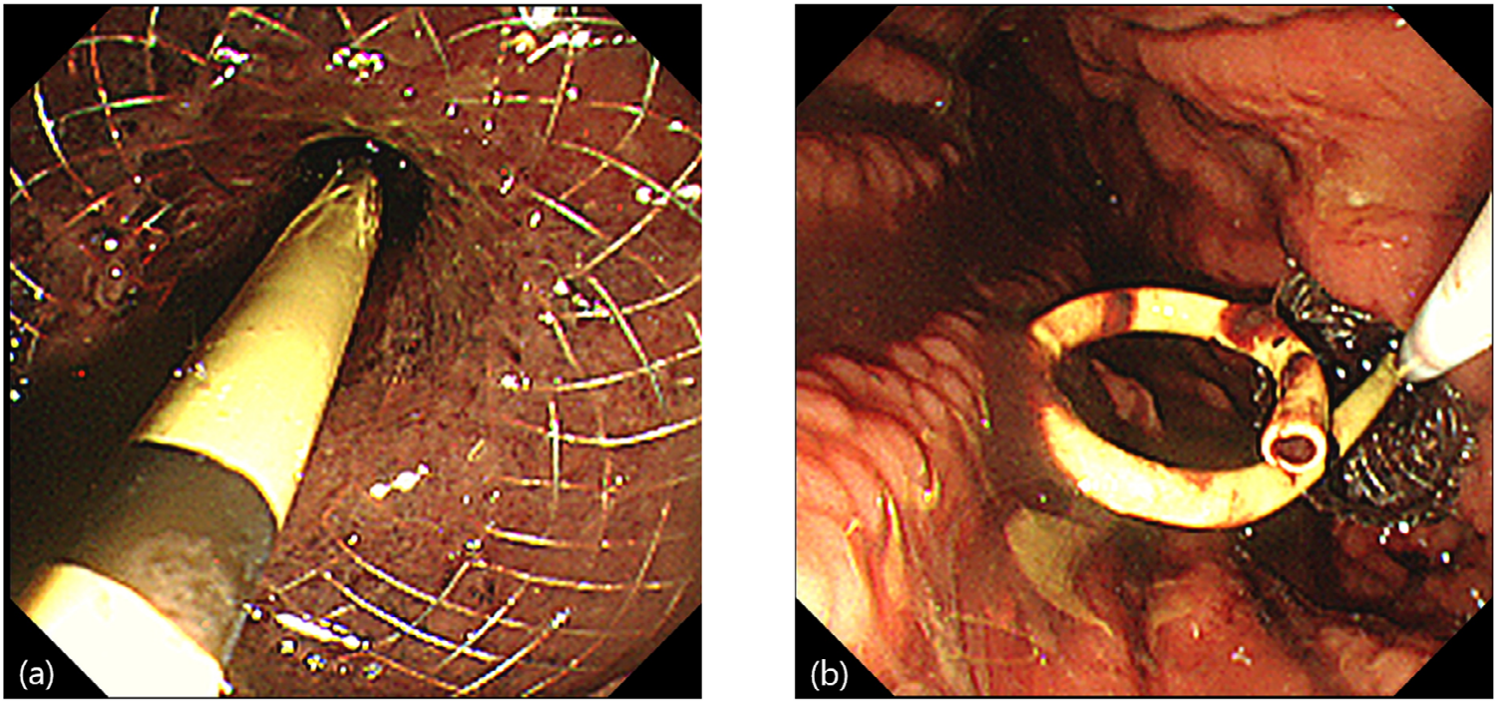
Insertion of a coaxial double‐pigtail plastic stent into lumen‐apposing metal stents (LAMS) after LAMS placement. (a) Endoscopic findings on insertion of the coaxial plastic stent within LAMS after LAMS placement for the treatment of patients with pancreatic fluid collections (PFCs). (b) Endoscopic findings for the placement of a coaxial double‐pigtail plastic stent (DPS) to LAMS after LAMS placement for the treatment of patients with PFCs to prevent mechanical abrasion/impingement of the adjacent vasculature by the distal flanges of the LAMS upon resolution of the PFCs.

Although the conventional DPS has been used as the primary drainage for the treatment of PFCs, the use of LAMS has recently increased. LAMS offers potential benefits such as easier DEN and reduced risks of perforation, stent occlusion, and stent migration compared with DPS. In addition, the placement of LAMS for patients with PFCs is technically easier and faster than with DPS, because the availability of a cautery tip for LAMS insertion allows placement without the need for initial needle puncture, wire guidance, or tract dilation, all of which require device exchange procedures. This might explain the significantly shorter procedural time observed in our study and several previous investigations [20, 21, 22, 23, 24, 25]. Additional advantages of LAMS over DPS and naso‐cystic tubes include its use in ICU patients without fluoroscopy during placement, its ability to be internalized, and its ease of use in additional treatments, including DEN [26]. Whereas DEN using DPS and naso‐cystic tubes is difficult and cumbersome, requiring stent removal and tract dilation. Several retrospective studies have shown that LAMS leads to high rates of clinical success and shorter procedure time and lower rates of recurrence and AEs than DPS, suggesting LAMS as the preferred stent [25, 27]. However, recent randomized controlled trials have shown that LAMS and DPS demonstrate similar outcomes regarding resolution of PFCs, DEN necessity, AEs, recurrence of PFCs, and cost‐effectiveness [28, 29]. In this study, we also experienced a need for DEN after LAMS placement and AEs such as spontaneous stent migration and delayed bleeding, but no instances of PFC recurrence were seen. We hypothesized that the cases of delayed bleeding after LAMS placement were well controlled of pancreatitis, but suddenly had hematemesis and lost consciousness, so likely secondary to mechanical impingement of adjacent vasculature by the distal flanges of the LAMS upon resolution of the PFCs [30]. We have therefore recently been attempting to add a coaxial DPS to LAMS to prevent mechanical impingement of adjacent vasculature by the distal flanges of the LAMS upon the resolution of PFCs (Figure 5). Indeed, a previous comparative retrospective study suggested that the addition of a coaxial DPS to LAMS may be associated with fewer AEs [31, 32, 33]. In any case, although LAMS appear to be optimal stents for EUS‐TD, few studies have clarified long‐term outcomes with the use of LAMS. The benefits of LAMS thus remain unclear, and optimal stent selection in EUS‐TD requires future prospective studies to further evaluate safety and long‐term outcomes.

One limitation of this study was that the design was a three‐center retrospective study, and the sample size cannot be considered sufficient. Some inherent biases are associated with retrospective studies, so the background characteristics of patients cannot be examined in detail.

In conclusion, our data suggest that EUS‐TD using LAMS for the treatment of PFCs is a simple, clinically effective, highly safe, and efficient treatment. Further randomized, prospective controlled trials are now needed to validate indications for EUS‐TD using LAMS for the treatment of PFCs.

## Author Contributions

*Conceptualization*: Keisuke Kinoshita, Kazuhisa Okamoto and Satoshi Fukuchi. *Formal analysis and investigation*: Keisuke Kinoshita, Kazuhisa Okamoto and Satoshi Fukuchi. *Writing—original draft preparation*: Keisuke Kinoshita, Kazuhisa Okamoto and Satoshi Fukuchi. *Writing—review and editing*: Ryota Sagami, Haruna Noguchi, Hidetoshi Akiyama, Yoshifumi Azuma, Yasuhisa Hiroshima, Takafumi Fuchino, Sotaro Ozaka, Takuro Uchida, Yuka Hirashita, Kensuke Fukuda, Ryo Ogawa, Kazuhiro Mizukami, Masaaki Kodama and Kazunari Murakami. *Resources*: Kazuhisa Okamoto, Satoshi Fukuchi and Mitsuteru Motomura. *Supervision*: Kazunari Murakami. All authors have approved the final version of the paper.

## Ethics Statement

This study was conducted in accordance with the principles of the Declaration of Helsinki and approved by the ethics committee at Oita University Hospital (approval no. 2773).

## Consent

Written informed consent was obtained from all patients for publication.

## Conflicts of Interest

The authors declare no conflicts of interest.

## Funding

The author has nothing to report.

## Clinical Trial Registration

Not applicable.

## Data Availability

The data that support the findings of this study are available from the corresponding author upon reasonable request.

## References

[1] Y. Nakai , H. Shiomi , T. Hamada , et al., “Early versus Delayed Interventions for Necrotizing Pancreatitis: A Systematic Review and Meta‐analysis,” DEN Open 3 (2023): e171. 36247314 10.1002/deo2.171 PMC9549879

[2] N. Oblizajek , N. Takahashi , S. Agayeva , et al., “Outcomes of Early Endoscopic Intervention for Pancreatic Necrotic Collections: A Matched Case‐control Study,” Gastrointestinal Endoscopy 91 (2020): 1303–1309. 31958461 10.1016/j.gie.2020.01.017

[3] C. Fabbri , C. Luigiano , and M. Marsico , “A Rare Adverse Event Resulting From the Use of a Lumen‐apposing Metal Stent for Drainage of a Pancreatic Fluid Collection: “the Buried Stent”,” Gastrointestinal Endoscopy 82 (2015): 585–587. 26279357 10.1016/j.gie.2015.04.035

[4] T. C. Seerden and F. P. Vleggaar , “Endoscopic Removal of Buried Lumen‐apposing Metal Stents Used for Cystogastrostomy and Cholecystogastrostomy,” Endoscopy 48 (2016): E179. 27213969 10.1055/s-0042-107073

[5] A. Y. Altonbary and H. Hakim , “The Buried Stent: A Rare Complication of Endoscopic Ultrasound‐guided Pancreatic Necrosectomy Using a Lumen‐apposing Metal Stent,” Endoscopy 49 (2017): E84–E85. 28142159 10.1055/s-0042-124505

[6] R. Sanchez‐Ocana , I. Peňas‐Herrero , and F. Santons‐Santamarta , “EUS‐guided Removal of a Buried Lumen‐apposing Metal Stent Caused by Delayed Inward Migration After Cyst‐gastrostomy,” Gastrointestinal Endoscopy 86 (2017): 229. 28351687 10.1016/j.gie.2017.03.023

[7] J. Y. Bang , M. Hasan , and U. Navaneethan , “Lumen‐apposing Metal Stents (LAMS) for Pancreatic Fluid Collection (PFC) Drainage: May Not be Business as Usual,” Gut 66 (2016): 2054–2056. 27582509 10.1136/gutjnl-2016-312812 PMC5749339

[8] J. Biedermann , S. Zeissig , S. Brückner , and J. Hampe , “EUS‐guided Stent Removal in Buried Lumen‐apposing Metal Stent Syndrome: A Case Series,” VideoGIE 5 (2020): 37–40. 31922082 10.1016/j.vgie.2019.09.002 PMC6945230

[9] S. Varadarajulu , J. Y. Bang , and B. S. Sutton , “Equal Efficacy of Endoscopic and Surgical Cystogastrostomy for Pancreatic Pseudocyst Drainage in a Randomized Trial,” Gastroenterology 145 (2013): 583–590. 23732774 10.1053/j.gastro.2013.05.046

[10] J. Y. Bang , J. P. Arnoletti , B. A. Holt , et al., “An Endoscopic Transluminal Approach, Compared with Minimally Invasive Surgery, Reduces Complications and Costs for Patients with Necrotizing Pancreatitis,” Gastroenterology 156 (2019): 1027–1040.e3. 30452918 10.1053/j.gastro.2018.11.031

[11] S. van Brunschot , J. van Grinsven , H. C. van Santvoort , et al., “Endoscopic or Surgical Step‐up Approach for Infected Necrotising Pancreatitis: A Multicentre Randomised Trial,” Lancet 391 (2018): 51–58. 29108721 10.1016/S0140-6736(17)32404-2

[12] S. Mukai , T. Itoi , T. H. Baron , et al., “Endoscopic Ultrasound‐guided Placement of Plastic vs. Biflanged Metal Stents for Therapy of Walled‐off Necrosis: A Retrospective Single‐center Series,” Endoscopy 47 (2015): 47–55. 25264765 10.1055/s-0034-1377966

[13] S. Mukai , T. Tsuchiya , T. Itoi , et al., “Prospective Evaluation of a New Biflanged Metal Stent for the Treatment of Pancreatic Fluid Collections (With videos),” Gastrointestinal Endoscopy 86 (2017): 203–207. 27908599 10.1016/j.gie.2016.11.025

[14] N. Fujimori , T. Osoegawa , A. Aso , et al., “Efficacy of Early Endoscopic Ultrasound‐Guided Transluminal Drainage for Postoperative Pancreatic Fistula,” Canadian Journal of Gastroenterology & Hepatology 2021 (2021): 6691705. 33564656 10.1155/2021/6691705 PMC7850853

[15] M. Tsujimae , H. Shiomi , A. Sakai , et al., “Computed Tomography Imaging‐based Predictors of the Need for a Step‐up Approach After Initial Endoscopic Ultrasound‐guided Transmural Drainage for Pancreatic Fluid Collections,” Surgical Endoscopy 37 (2023): 1096–1106. 36123547 10.1007/s00464-022-09610-2

[16] K. F. Binmoeller and J. Shah , “A Novel Lumen‐apposing Stent for Transluminal Drainage of Nonadherent Extraintestinal Fluid Collections,” Endoscopy 43 (2011): 337–342. 21264800 10.1055/s-0030-1256127

[17] D. E. Penn , P. V. Draganov , M. S. Wagh , C. E. Forsmark , A. R. Gupte , and S. S. Chauhan , “Prospective Evaluation of the Use of Fully Covered Self‐expanding Metal Stents for EUS‐guided Transmural Drainage of Pancreatic Pseudocysts,” Gastrointestinal Endoscopy 76 (2012): 679–684. 22732874 10.1016/j.gie.2012.04.457

[18] D. Yang , Y. B. Perbtani , L. K. Mramba , et al., “Safety and Rate of Delayed Adverse Events With Lumen‐apposing Metal Stents (LAMS) for Pancreatic Fluid Collections: A Multicenter Study,” Endoscopy International Open 6 (2018): E1267–E1275. 30302385 10.1055/a-0732-502 PMC6175687

[19] M. Kumar , U. Sonika , S. Sachdeva , et al., “Natural History of Asymptomatic Walled‐off Necrosis in Patients with Acute Pancreatitis,” Cureus 15 (2023): e34646. 36895535 10.7759/cureus.34646 PMC9990741

[20] J. B. Gornals , C. De la Serna‐Higuera , A. Sánchez‐Yague , C. Loras , A. M. Sánchez‐Cantos , and M. Pérez‐Miranda , “Endosonography‐guided Drainage of Pancreatic Fluid Collections With a Novel Lumen‐apposing Stent,” Surgical Endoscopy 27 (2013): 1428–1434. 23232994 10.1007/s00464-012-2591-y

[21] S. Khan , S. Chandran , J. Chin , et al., “Drainage of Pancreatic Fluid Collections Using a Lumen‐apposing Metal Stent With an Electrocautery‐enhanced Delivery System,” Journal of Gastroenterology and Hepatology 36 (2021): 3395–3401. 34370869 10.1111/jgh.15658

[22] D. Oh , J. H. Lee , T. J. Song , et al., “Clinical Outcomes of EUS‐guided Transluminal Drainage With a Novel Lumen‐apposing Metal Stent for Postoperative Pancreatic Fluid Collection After Pancreatic Surgery,” Gastrointestinal Endoscopy 95 (2022): 735–746. 34971669 10.1016/j.gie.2021.12.015

[23] N. L. H. Bekkali , M. K. Nayar , J. S. Leeds , R. M. Charnley , M. T. Huggett , and K. W. Oppong , “A Comparison of Outcomes Between a Lumen‐apposing Metal Stent With Electrocautery‐enhanced Delivery System and a bi‐flanged Metal Stent for Drainage of Walled‐off Pancreatic Necrosis,” Endoscopy International Open 5 (2017): E1189–E1196. 29202002 10.1055/s-0043-120831 PMC5698007

[24] A. A. Siddiqui , T. E. Kowalski , D. E. Loren , et al., “Fully Covered Self‐expanding Metal Stents versus Lumen‐apposing Fully Covered Self‐expanding Metal Stent versus Plastic Stents for Endoscopic Drainage of Pancreatic Walled‐off Necrosis: Clinical Outcomes and Success,” Gastrointestinal Endoscopy 85 (2017): 758–765. 27566053 10.1016/j.gie.2016.08.014

[25] Y. I. Chen , J. Yang , S. Friedland , et al., “Lumen Apposing Metal Stents Are Superior to Plastic Stents in Pancreatic Walled‐off Necrosis: A Large International Multicenter Study,” Endoscopy International Open 7 (2019): E347–E354. 30834293 10.1055/a-0828-7630 PMC6395102

[26] J. Yoo , L. Yan , R. Hasan , et al., “Feasibility, Safety, and Outcomes of a Single‐step Endoscopic Ultrasonography‐guided Drainage of Pancreatic Fluid Collections Without Fluoroscopy Using a Novel Electrocautery‐enhanced Lumen‐apposing, Self‐expanding Metal Stent,” Endoscopy and Ultrasound 6 (2017): 131–135. 10.4103/2303-9027.204814 PMC5418966 28440239

[27] A. Bapaye , N. A. Dubale , K. A. Sheth , et al., “Endoscopic Ultrasonography‐guided Transmural Drainage of Walled‐off Pancreatic Necrosis: Comparison Between a Specially Designed Fully Covered bi‐flanged Metal Stent and Multiple Plastic Stents,” Digestive Endoscopy 29 (2017): 104–110. 27463528 10.1111/den.12704

[28] J. Y. Bang , U. Navaneethan , M. K. Hasan , B. Sutton , R. Hawes , and S. Varadarajulu , “Non‐superiority of Lumen‐apposing Metal Stents Over Plastic Stents for Drainage of Walled‐off Necrosis in a Randomised Trial,” Gut 68 (2019): 1200–1209. 29858393 10.1136/gutjnl-2017-315335 PMC6582745

[29] L. Boxhoorn , R. C. Verdonk , M. G. Besselink , et al., “Comparison of Lumen‐apposing Metal Stents Versus Double‐pigtail Plastic Stents for Infected Necrotising Pancreatitis,” Gut 72 (2023): 66–72. 35701094 10.1136/gutjnl-2021-325632

[30] K. Keisuke , K. Mizukami , K. Fukuda , et al., “Pancreatic Cancer With Pseudoaneurysm After Duckbill‐shaped Anti‐reflux Metal Stent Placement: A Case Report,” DEN Open 3 (2022): e203. 36568965 10.1002/deo2.203 PMC9768111

[31] M. Puga , C. F. Consiglieri , J. Busquets , et al., “Safety of Lumen‐apposing Stent With or Without Coaxial Plastic Stent for Endoscopic Ultrasound‐guided Drainage of Pancreatic Fluid Collections: A Retrospective Study,” Endoscopy 50 (2018): 1022–1026. 29590668 10.1055/a-0582-9127

[32] P. Vanek , P. Falt , P. Vitek , et al., “EUS‐guided Transluminal Drainage Using Lumen‐apposing Metal Stents With or Without Coaxial Plastic Stents for Treatment of Walled‐off Necrotizing Pancreatitis: A Prospective Bicentric Randomized Controlled Trial,” Gastrointestinal Endoscopy 97 (2023): 1070–1080. 36646148 10.1016/j.gie.2022.12.026

[33] J. AbiMansour , V. Jaruvongvanich , S. Velaga , et al., “Coaxial Plastic Stent Placement Within Lumen‐apposing Metal Stents for the Management of Pancreatic Fluid Collections: A Systemic Review and Meta‐analysis,” Clinical Endoscopy 57 (2024): 595–603. 39044669 10.5946/ce.2023.297 PMC11474481

